# Meta-analysis of genome-wide association studies identifies common susceptibility polymorphisms for colorectal and endometrial cancer near *SH2B3* and *TSHZ1*

**DOI:** 10.1038/srep17369

**Published:** 2015-12-01

**Authors:** Timothy HT Cheng, Deborah Thompson, Jodie Painter, Tracy O’Mara, Maggie Gorman, Lynn Martin, Claire Palles, Angela Jones, Daniel D. Buchanan, Aung Ko Win, John Hopper, Mark Jenkins, Noralane M. Lindor, Polly A. Newcomb, Steve Gallinger, David Conti, Fred Schumacher, Graham Casey, Graham G Giles, Paul Pharoah, Julian Peto, Angela Cox, Anthony Swerdlow, Fergus Couch, Julie M Cunningham, Ellen L Goode, Stacey J Winham, Diether Lambrechts, Peter Fasching, Barbara Burwinkel, Hermann Brenner, Hiltrud Brauch, Jenny Chang-Claude, Helga B. Salvesen, Vessela Kristensen, Hatef Darabi, Jingmei Li, Tao Liu, Annika Lindblom, Per Hall, Magdalena Echeverry de Polanco, Monica Sans, Angel Carracedo, Sergi Castellvi-Bel, Augusto Rojas-Martinez, Samuel Aguiar Jnr, Manuel R. Teixeira, Alison M Dunning, Joe Dennis, Geoffrey Otton, Tony Proietto, Elizabeth Holliday, John Attia, Katie Ashton, Rodney J Scott, Mark McEvoy, Sean C Dowdy, Brooke L Fridley, Henrica MJ Werner, Jone Trovik, Tormund S Njolstad, Emma Tham, Miriam Mints, Ingo Runnebaum, Peter Hillemanns, Thilo Dörk, Frederic Amant, Stefanie Schrauwen, Alexander Hein, Matthias W Beckmann, Arif Ekici, Kamila Czene, Alfons Meindl, Manjeet K Bolla, Kyriaki Michailidou, Jonathan P Tyrer, Qin Wang, Shahana Ahmed, Catherine S Healey, Mitul Shah, Daniela Annibali, Jeroen Depreeuw, Nada A. Al-Tassan, Rebecca Harris, Brian F. Meyer, Nicola Whiffin, Fay J Hosking, Ben Kinnersley, Susan M. Farrington, Maria Timofeeva, Albert Tenesa, Harry Campbell, Robert W. Haile, Shirley Hodgson, Luis Carvajal-Carmona, Jeremy P. Cheadle, Douglas Easton, Malcolm Dunlop, Richard Houlston, Amanda Spurdle, Ian Tomlinson

**Affiliations:** 1 Molecular and Population Genetics Laboratory, Wellcome Trust Centre for Human Genetics, University of Oxford, Roosevelt Drive, Oxford OX3 7BN, UK; 2 Centre for Cancer Genetic Epidemiology, Public Health and Primary Care, University of Cambridge, Cambridge CB1 8RN, UK; 3 The Molecular Cancer Epidemiology Laboratory, QIMR Berghofer Medical Research Institute, Brisbane 4006, Australia; 4 Oncogenomics Group, Genetic Epidemiology Laboratory, Department of Pathology, The University of Melbourne, Victoria, Australia; 5 Centre for Epidemiology and Biostatistics, The University of Melbourne, Victoria, Australia; 6 Department of Health Sciences Research, Mayo Clinic, Scottsdale, AZ, USA; 7 Cancer Prevention Program, Fred Hutchinson Cancer Research Center, Seattle, WA, USA; 8 Lunenfeld-Tanenbaum Research Institute, Mount Sinai Hospital, Toronto, ON, Canada; 9 Department of Preventive Medicine, University of Southern California, Los Angeles, CA, USA; 10 Cancer Epidemiology Centre, Cancer Council Victoria, Melbourne, Australia; 11 Department of Epidemiology and Preventive Medicine, Monash University, Melbourne, Australia; 12 Centre for Cancer Genetic Epidemiology, Department of Oncology, University of Cambridge, Cambridge, UK; 13 London School of Hygiene and Tropical Medicine, London, UK; 14 Sheffield Cancer Research Centre, Department of Oncology, University of Sheffield, Sheffield, UK; 15 Division of Genetics and Epidemiology, Institute of Cancer Research, Sutton, UK and 17 Division of Breast Cancer Research, Institute of Cancer Research, London, UK; 16 Department of Laboratory Medicine and Pathology, Mayo Clinic, Rochester, MN, USA; 17 Department of Health Sciences Research, Mayo Clinic, Rochester, MN, USA; 18 Department of Oncology, KU Leuven, Belgium; 19 University of California at Los Angeles, Department of Medicine, Division of Hematology/Oncology, David Geffen School of Medicine, Los Angeles, CA, USA; 20 Department of Gynecology and Obstetrics, University Hospital Erlangen, Friedrich-Alexander University Erlangen-Nuremberg, Erlangen, Germany; 21 Division of Cancer Epidemiology, German Cancer Research Center, Heidelberg, Germany; 22 Dr. Margarete Fischer-Bosch-Institute of Clinical Pharmacology Stuttgart, University of Tuebingen, Germany; 23 Division of Cancer Epidemiology, German Cancer Research Center, Heidelberg, Germany; 24 Department of Clinical Science, Center for Cancer Biomarkers, University of Bergen, Norway; 25 Department of Genetics, Institute for Cancer Research, The Norwegian Radium Hospital, Oslo, Norway; The K.G. Jebsen Center for Breast Cancer Research, Institute for Clinical Medicine, Faculty of Medicine, University of Oslo, Oslo, Norway; Department of Clinical Molecular Oncology, Division of Medicine, Akershus University Hospital, Ahus, Norway; 26 Department of Molecular Medicine and Surgery, Karolinska Institutet, Stockholm, Sweden; 27 Department of Medical Epidemiology and Biostatistics, Karolinska Institutet, Stockholm, Sweden; 28 Grupo de investigación Citogenética, Filogenia y Evolución de Poblaciones, Universidad del Tolima, Ibagué, Tolima, Colombia; 29 Departamento de Antropologia Biologica, Facultad de Humanidades, UDELAR, Magallanes 1577, CP 11200, Montevideo, Uruguay; 30 Universidade de Santiago de Compostel, R/ San Francisco s/n 15782, Santiago de Compostela, Spain; 31 Genetic Predisposition to Colorectal Cancer Group, Gastrointestinal & Pancreatic Oncology Team, IDIBAPS/CIBERehd/Hospital Clínic, Centre Esther Koplowitz (CEK), Rosselló 153 planta 4, 08036 Barcelona, Spain; 32 Universidad Autónoma De Nuevo León, Pedro de Alba s/n, San Nicolás de Los Garza, Nuevo León, Mexico; 33 Hospital A.C. Camargo, São Paulo, Brazil; 34 Department of Genetics and IPO-Porto Research Center (CI-IPOP), Portuguese Oncology Institute of Porto (IPO-Porto), Porto, Portugal, and Biomedical Sciences Institute (ICBAS), University of Porto, Porto, Portugal; 35 School of Medicine and Public Health, University of Newcastle, NSW, Australia; 36 Hunter Medical Research Institute, John Hunter Hospital, Newcastle, NSW, Australia; 37 Centre for Clinical Epidemiology and Biostatistics, School of Medicine and Public Health, University of Newcastle, NSW, Australia; 38 Department of Obstetrics and Gynecology, Division of Gynecologic Oncology, Mayo Clinic, Rochester, MN, USA; 39 Department of Biostatistics, University of Kansas Medical Center, Kansas City, KS, USA; 40 Centre for Cancer Biomarkers, Department of Clinical Science, The University of Bergen, Norway; 41 Department of Women’s and Children’s Health, Karolinska Institutet, Karolinska University Hospital, Stockholm, Sweden; 42 Department of Gynaecology, Jena University Hospital - Friedrich Schiller University, Jena, Germany; 43 Hannover Medical School, Clinics of Gynaecology and Obstetrics, Hannover, Germany; 44 Hannover Medical School, Gynaecology Research Unit, Hannover, Germany; 45 Division of Gynaecological Oncology, University Hospital Leuven, Leuven, Belgium; 46 Institute of Human Genetics, University Hospital Erlangen, Friedrich-Alexander University Erlangen-Nuremberg, Erlangen, Germany; 47 Department of Obstetrics and Gynecology, Division of Tumor Genetics, Technical University of Munich, Munich, Germany; 48 Department of Obstetrics and Gynecology, Division of Gynecologic Oncology, University Hospitals, KU Leuven, University of Leuven, 3000, Belgium; 49 Vesalius Research Center, Leuven, 3000, Belgium; 50 Department of Genetics, King Faisal Specialist Hospital and Research Center, P.O.Box 3354, Riyadh11211, Saudi Arabia; 51 Institute of Cancer and Genetics, School of Medicine, Cardiff University, Heath Park, Cardiff, CF14 4XN, UK; 52 Division of Genetics and Epidemiology, The Institute of Cancer Research, Sutton, Surrey SM2 5NG, UK; 53 Colon Cancer Genetics Group, Institute of Genetics and Molecular Medicine, University of Edinburgh and MRC Human Genetics Unit, Western General Hospital Edinburgh, Crewe Road, Edinburgh, EH4 2XU, UK; 54 The Roslin Institute, University of Edinburgh, Easter Bush, Roslin, EH25 9RG, UK; 55 Centre for Population Health Sciences, University of Edinburgh, Edinburgh, EH8 9AG, UK; 56 Stanford Cancer Institute, Lorry Lokey Building/SIM 1, 265 Campus Drive, Ste G2103, Stanford, CA 94305-5456, USA; 57 Department of Cancer Genetics, St. George’s University of London, London SW17 0RE, UK; 58 Genome Center and Department of Biochemistry and Molecular Medicine, School of Medicine, University of California, Davis, USA; 59 Oxford NIHR Comprehensive Biomedical Research Centre, Wellcome Trust Centre for Human Genetics, Roosevelt Drive, Oxford OX3 7BN, UK

## Abstract

High-risk mutations in several genes predispose to both colorectal cancer (CRC) and endometrial cancer (EC). We therefore hypothesised that some lower-risk genetic variants might also predispose to both CRC and EC. Using CRC and EC genome-wide association series, totalling 13,265 cancer cases and 40,245 controls, we found that the protective allele [G] at one previously-identified CRC polymorphism, rs2736100 near *TERT*, was associated with EC risk (odds ratio (OR) = 1.08, P = 0.000167); this polymorphism influences the risk of several other cancers. A further CRC polymorphism near *TERC* also showed evidence of association with EC (OR = 0.92; P = 0.03). Overall, however, there was no good evidence that the set of CRC polymorphisms was associated with EC risk, and neither of two previously-reported EC polymorphisms was associated with CRC risk. A combined analysis revealed one genome-wide significant polymorphism, rs3184504, on chromosome 12q24 (OR = 1.10, P = 7.23 × 10^−9^) with shared effects on CRC and EC risk. This polymorphism, a missense variant in the gene *SH2B3*, is also associated with haematological and autoimmune disorders, suggesting that it influences cancer risk through the immune response. Another polymorphism, rs12970291 near gene *TSHZ1*, was associated with both CRC and EC (OR = 1.26, P = 4.82 × 10^−8^), with the alleles showing opposite effects on the risks of the two cancers.

Colorectal carcinoma (CRC) is the fourth commonest cancer in the western world and cancer of the uterine corpus, or endometrial carcinoma (EC), is the fourth commonest cancer among women. Both cause significant morbidity and mortality worldwide. There is evidence from rare, Mendelian cancer predisposition syndromes that CRC and EC can have a common aetiology. Specifically, germline mutations in mismatch repair (MMR) genes *MLH1*, *MSH2*, *MSH6* and *PMS2*^1^, and in DNA polymerases *POLD1* and *POLE*^2^ predispose to a high incidence (lifetime risk 30–71%^2^,^3^,^4^,^5^) of both CRC and EC. The MMR system maintains genomic stability by correcting mismatched nucleotide pairs that arise during DNA replication and MMR mutations cause a microsatellite instability (MSI+) phenotype in CRCs and ECs^6^. Bi-allelic *MLH1* promoter methylation^7^,^8^ and a few somatic mutations in *MLH1* and *MSH2*^9^ are seen in sporadic CRCs and ECs, causing the same MSI+ and hypermutator phenotype. Histologically, MMR-deficient CRCs and ECs are characterised by poor differentiation and the presence of mucinous and signet-cell features and tumour-infiltrating lymphocytes^10^,^11^. *POLE* and *POLD1* encode polymerases that synthesise respectively the leading and lagging strand of the DNA replication fork. The exonuclease (proofreading) domains of these polymerases increase replication fidelity by recognising and excising mispaired bases^12^,^13^. Germline missense mutations in the exonuclease domains of *POLD1* and *POLE* predispose to both CRC and EC, and somatic *POLE* mutations occur in sporadic CRCs and ECs^2^,^14^,^15^,^16^. Polymerase exonuclease domain mutations (EDMs) do not cause MSI, but lead to an ultramutator phenotype, with over one million base substitutions in some cancers.

Genome-wide association studies (GWAS) have successfully identified tens of common single nucleotide polymorphisms (SNPs) associated with a modestly increased risk (typically 10–25%) of CRC. In addition, one EC SNP, near *HNF1B*, has been reported at stringent levels of statistical significance. To date, the lists of CRC and EC SNPs are non-overlapping. Since CRC and EC may share mechanisms of pathogenesis, as evidenced by the high-penetrance germline mutations and the somatic (epi)mutations discussed above, we hypothesised (i) that some CRC SNPs may predispose to EC, and *vice versa*, and (ii) that there exist unidentified SNPs that predispose to both CRC and EC. In this study, we tested these hypotheses using 16 different CRC and EC GWAS data sets, totalling 13,265 cancer cases and 40,245 cancer-free or population controls.

## Methods

### GWAS data sets

Five CRC GWAS data sets genotyped on various Illumina tag-SNP arrays were available, comprising: (i) CORGI (UK1), (ii) Scotland 1, (iii) VICTOR/QUASAR2/BC58, (iv) CFR1 and (v) CFR2/CGEMS (total 5,725 cases and 6,671 controls)^17^,^18^,^19^,^20^,^21^. The VQ58, CORGI and Scotland 1 series were genotyped using Illumina Hap300, Hap240S, Hap370, Hap550 or Omni2.5M arrays. BC58 genotyping was performed as part of the WTCCC2 study on Hap1.2M-Duo Custom arrays. The CCFR samples were genotyped using Illumina Hap1M, Hap1M-Duo or Omni-express arrays. CGEMS samples (all controls) were genotyped using Illumina Hap300 and Hap240 or Hap550 arrays. Standard quality -control measures were applied as reported^17^. Moreover, any duplicate or cryptically related samples were excluded by pairwise identity by descent (IBD) analysis.

EC GWAS comprised: (i) NSECG, (ii) ANECS and (iii) SEARCH (total 2,212 cases and 6,725 controls)^22^. All samples were of European ancestry with the majority of samples from the UK, and others from USA and Australia. Standard quality control measures were performed for each GWAS, as described in the referenced publications, and details about each dataset are shown in Table 1. Some of the control datasets, including the Wellcome Trust Case Control Consortium 2 (WTCCC2)^23^, have previously been used in both CRC and EC GWAS. We ensured that such controls were assigned proportionately to case data sets and were not used more than once (Table 1).

Principal component analysis (PCA) was conducted for all samples together, to ensure that all individuals were of European ancestry and we excluded all individuals who clustered outside the main centroid in pairwise plots of the first 4 PCs. The adequacy of case-control matching and possibility of differential genotyping of cases and controls was assessed using Q-Q plots of test statistics. λ_GC_ values for the CORGI, Scotland1, VQ58, CCFR1 and CCFR2 studies were 1.02, 1.01, 1.01, 1.02 and 1.03 respectively, and those for NSECG, ANECS and SEARCH were 1.02, 1.02 and 1.00 respectively.

### EC targeted genotyping data sets

A further 4,330 EC cases and 26,849 female controls were genotyped as part of the Endometrial Cancer Association Consortium (ECAC), with samples from seven countries: UK, USA, Belgium, Germany, Norway, Sweden and Australia. The controls were selected from healthy females participating in the Breast Cancer Association Consortium (BCAC) and Ovarian Cancer Association Consortium (OCAC) part of the iCOGS project and matched and analysed with cases in eight groups by geographical location (see Table 1). These samples were genotyped using a custom Illumina Infinium iSelect array with 211,155 SNPs designed by the COGS (Collaborative Oncological Gene-environment Study) initiative^24^,^25^,^26^,^27^. The SNPs on this array were chosen based on regions of interest from previous breast, prostate, ovarian and endometrial cancer studies, rather than on genome-wide coverage. We did not impute genotypes from the COGS studies, but included directly-genotyped SNPs in the discovery meta-analysis. These SNPs were not used for locus fine mapping.

### Association study and meta-analysis

Whole-genome imputation using two reference panels (1000 Genomes 2012 release^28^ and 196 high-coverage whole genome-sequenced UK individuals) was performed with IMPUTE2^29^, yielding up to 6 million SNPs either typed or imputed with high quality (info score >0.9). Case-control analysis for each GWAS data set was performed using frequentist tests with a logistic regression model using SNPTEST (v2.4)^30^. There was no evidence of systematic over-dispersion of the test statistic for any of the 16 studies (lambda_GC_ = 1.01–1.04 based on weakly correlated SNPs, r^2^ < 0.2). Fixed-effects, inverse variance weighted meta-analysis was conducted for the 6 million well-imputed SNPs in the eight CRC and EC GWAS (8,935 cases, 13,396 controls) across the genome using GWAMA (v2.1)^31^. For the ~200,00 SNPs genotyped on the COGS array, the additional 4,330 EC cases and 26,849 controls from ECAC were included in a meta-analysis of 16 studies yielding a total of 13,265 cases and 40,245 controls for these loci. SNPs with globally significant CRC/EC associations (P_meta_ < 5 × 10^−8^) were identified and the regions examined using standard fine mapping and annotation methods.

### Previously reported CRC and EC SNPs

The effects of 25 previously published tag-SNPs that have been formally associated with CRC risk in GWAS were investigated in EC (Table 2). We additionally assessed two SNPs (near *TERT*^32^ and *MTHFR*^33^,^34^) with convincing CRC associations from focussed studies. We estimated that our EC sample set provided 72% power to detect the effect of a typical CRC SNP (allele frequency = 0.25, per allele odds ratio = 1.1) at P = 0.05, and 23% power to detect a similar allele at P = 0.001, corresponding to a false discovery rate of q = 0.05 in our sample. Two EC SNPs from GWAS^22^ were similarly investigated in CRC. All of these SNPs were either discovered or replicated in European populations and were genotyped directly or had near-perfect proxies on the Illumina GWAS arrays used; 13 of the SNPs were also present on the iCOGS arrays. Three EC SNPs in the *TERT-CLPTM1L* region^35^ were not included in this analysis, owing to poor tagging on the GWAS arrays and hence sub-optimal imputation.

### Genome-wide enrichment of susceptibility SNPs between CRC and EC

Beyond the 29 previously published associations, we investigated the presence of genome-wide enrichment for CRC and EC. After removing previous associations, we pruned the set of 6 million typed or well-imputed SNPs (*r*^*2*^ < 0.1) to 246,896. Using several P value thresholds, we determined whether there was a tendency for the same SNPs to co-occur in the lists of putative CRC and EC SNPs, irrespective of direction of effect.

## Results

We initially investigated the 29 previously-identified CRC and EC polymorphisms (Table 2). One SNP, rs2736100, originally reported in CRC^32^, was significantly associated with EC risk (OR: 0.93, 95% confidence interval (95% CI): 0.89-0.96, P = 0.000167) after correcting for multiple testing (P<0.001). The risk allele for CRC [A] was protective in EC. rs2736100 lies in the intronic region of the telomerase reverse transcriptase *TERT.* It or highly correlated SNPs have previously been associated with the risk of multiple different cancer types, and we ourselves have previously found evidence that these *TERT* SNPs are associated with EC risk^35^. Two other CRC SNPs (rs6691170 and rs10936599) were nominally associated with EC risk (P < 0.05). Interestingly, the latter of these lies close to the telomerase RNA component *TERC* locus; it is a multi-cancer risk SNP^36^,^37^,^38^ and has been associated with longer telomeres. Overall, 15 of the 29 SNPs showed the same direction of effect in both cancer types (that is, same nominal risk allele, irrespective of effect size), and this evidently was not a significant deviation from randomness (P = 1, binomial sign test).

**Figure 1:**
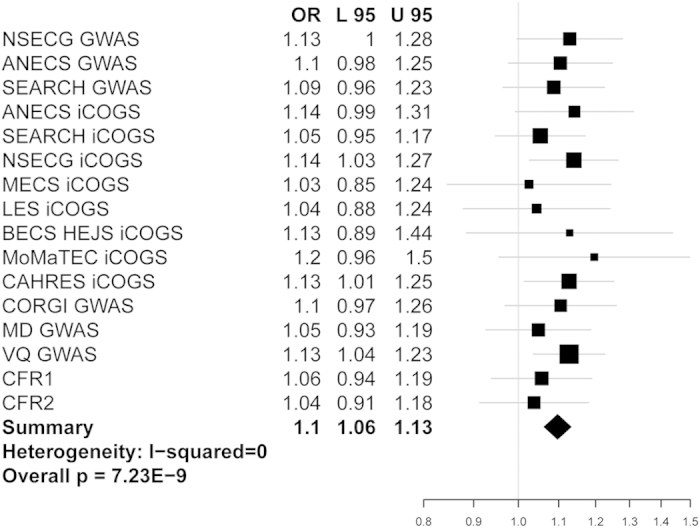
Forest plot showing association between cancer risk and rs3184504 genotype in each data set. Studies are shown in order of EC GWAS, EC iCOGS and CRC GWAS (Table 1). Black squares represent the point estimate of the odds ratio and have areas proportional to study size. Lines represent 95% confidence intervals. The diamond shows the summary statistic. The overall heterogeneity statistic is shown. There is also no evidence of heterogeneity between the pooled CRC and pooled EC studies (details not shown).

**Figure 2:**
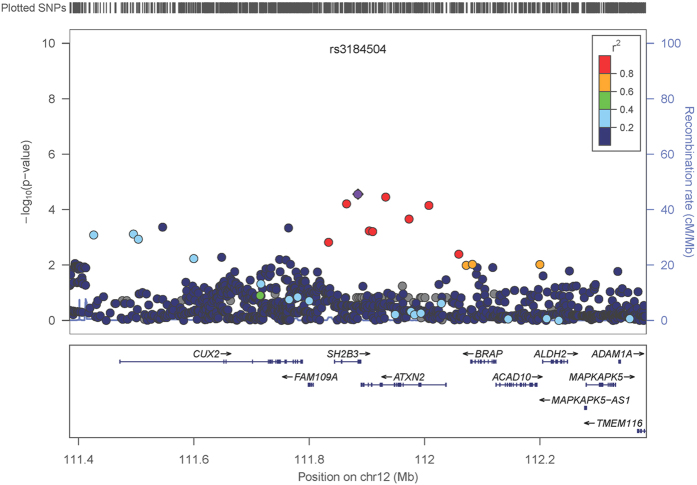
Regional association plot for region around rs3184504. Plots are produced in LocusZoom and show the most strongly associated SNP, rs3184504 (purple diamond). rs7137828, intron of *ATXN2*, is the SNP with the second lowest P value. The primary aim of this analysis is to compare association signals among SNPs in the region. Therefore, the data are derived from a meta-analysis of genotyped or high-quality imputed SNPs in the GWAS data sets, and because imputation quality was more variable in iCOGS than in the GWAS data, the iCOGS samples are not included.

Meta-analysis of all CRC and EC data sets revealed a single genome-wide significant SNP, rs3184504, on chromosome 12q24 (OR: 1.10, 95% CI 1.07–1.13, P_meta_: 7.23 × 10^−9^, heterogeneity *I*^*2*^ = 0; Fig. 1, Supplementary Table 1). This SNP is a missense variant (p.Trp262Arg) in exon 4 of *SH2B3*. It has not previously been associated with either CRC or EC. The major [C] allele was consistently the risk allele in all datasets, including those analysed using the iCOGS array, on which the SNP was included due to promising, but unproven, associations below genome-wide significance in previous breast cancer and EC GWAS. An additional 3 SNPs (Fig. 2) in strong pairwise linkage disequilibrium (LD) with rs3184504 (r^2^ > 0.9) showed strong evidence of CRC-EC association (P_fine mapping_ < 10^−5^). These 4 SNPs lie in a 68kb region, that includes the genes *SH2B3* and *ATXN2*, and their functional annotation is shown in Supplementary Table 2. None of the 4 SNPs was associated with the mRNA level of *SH2B3*, *ATXN2* or other nearby genes in public eQTL databases (details not shown).

**Figure 3:**
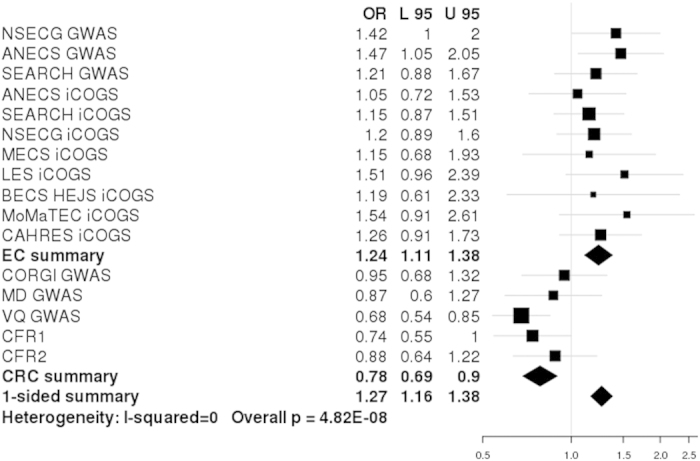
Forest plot showing association between cancer risk and rs12970291 genotype in each data set. Legend is as for Fig. 1.

**Figure 4:**
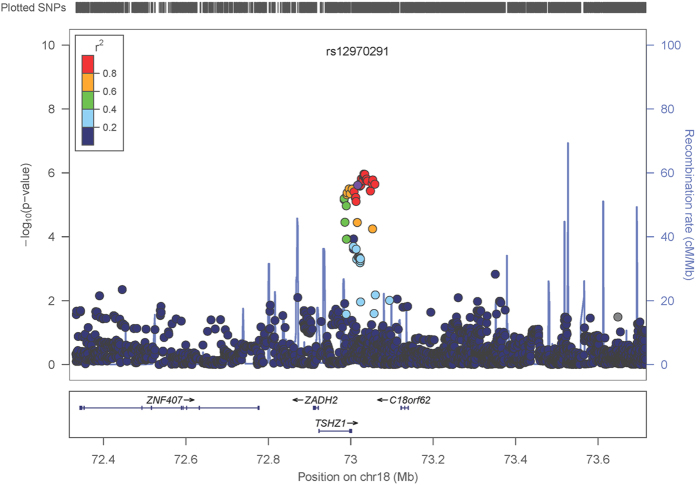
Regional association plot for region around rs12970291. Legend is as for Fig. 2, except as follows. The most strongly associated SNP from the full discovery meta-analysis (rs12970291, purple diamond) is not the most strongly associated in the GWAS data sets. The most strongly associated SNP, rs35185115, lies about 30kb downstream of *TSHZ1*, but this SNP imputed poorly in iCOGS and was therefore assessed in fewer samples in the discovery meta-analysis than rs12970291, which was directly genotyped in iCOGS.

There are SNPs that have previously been independently identified in GWAS of different phenotypes where the risk allele for one phenotype is the protective allele for another^39^,^40^. In order to search for SNPs for which the same allele has differing directions of effect in CRC and EC, we conducted a fixed-effect meta-analysis with the odds ratios of all the CRC SNPs GWAS inverted (Supplementary Table 3). In this analysis, we discovered rs12970291 on chromosome 18q22, where the major G allele is protective in CRC (OR:0.78, 95%CI:0.69-0.90, 3.42 × 10^−4^) and confers risk in EC (OR:1.24, 95%CI: 1.11–1.38, p:1.11 × 10^−4^). In meta-analysis, the rs12970291 association reached genome-wide significance (OR:1.26, 95%CI:1.16–1.38, P_meta_:4.82 × 10^−8^; Fig. 3). Fine mapping analysis identified a large number of SNPs in high pairwise LD with rs12970291 (r^2^ > 0.85), in a 70 kb region that includes the gene *TSHZ1*, which is ~15 kb proximal to rs12970291 (Fig. 4). Seventeen SNPs had a stronger disease association than rs12970291 in fine mapping, with the lowest P value at rs35185115 (P_fine mapping_ = 1.08 × 10^−6^). Fine mapping of CRC and EC GWAS separately (Supplementary Figure 1) showed an association peak occurring in the same LD block between 10.5–51.8 kb downstream of *TSHZ1*, while an additional suggestive association signal near rs17263435 (P_EC_ = 4.35 × 10^−5^) was not present in CRC (P_CRC_ = 0.1). Several SNPs in the region have potential functional importance (Supplementary Table 4), and of particular note is the missense SNP rs3390274 (p.Ala468Thr) in the last exon of *TSHZ1*. SNPs with a pairwise LD of >0.4 with rs12970291 in the region were not significantly associated with mRNA level of *TSHZ1* or other nearby genes in public eQTL databases (details not shown).

Finally, we performed genome-wide enrichment analysis for nearly 250,000 independent SNPs (*r*^*2*^ < 0.1) below genome-wide significance levels to investigate whether there was a set of cryptic shared CRC and EC risk loci (Supplementary Table 5). Using P value thresholds of 10^−3^, 10^−2^ and 0.05, we found no evidence of a significant sharing of CRC and EC SNPs using this method.

## Discussion

Using a combined CRC and EC GWAS meta-analysis, we have identified a region on chromosome 12q24.1 spanning two genes, *SH2B3* and *ATXN2*, which contains a SNP that is formally associated at GWAS thresholds of significance with cancer risk. Of the variants in this region, rs3184504 is of particular interest, because it is a non-synonymous change (TGG → CGG; p.Trp262Arg) in the pleckstrin homology domain of SH2B3, which is *a priori* a much stronger candidate than the spinocerebellar ataxia gene *ATXN2*. SH2B3 is a member of the SH2B adaptor family of proteins and is involved in a range of signalling activities by growth factor and cytokine receptors. It is a key negative regulator in cytokine signalling in haematopoiesis, and is expressed at a high level in the bone marrow and white blood cells, but at a low level in the normal bowel and endometrium (EMBL-EBI expression atlas). Comparative genomics shows that the rs3184504 risk allele (C, Arg residue) is conserved in all primates and some vertebrates (Supplementary Figure 1), and has a much lower allele frequency (~0.5) in Europeans than in African, Asian and admixed American populations (~1.0). Amino acids Trp (tryptophan) and Arg (arginine) present in the two forms of the polymorphic SH2B3 protein possess a hydrophobic (uncharged) and positively charged side chain respectively. Different programs that predict the effect of this variation on protein function vary in their assessment (Grantham score = 121 (range 0–215)^41^, Polyphen2 = 0.12^42^, SIFT = 1.0^43^, CADD score PHRED-scaled = 5.532^44^); overall, the possibility remains that the amino acid change has a modest or greater effect on protein function. The NHGRI GWAS Catalog shows that SNPs in the *SH2B3*/*ATNX2* region including rs3184504 and rs653178 have been previously associated with immune-mediated conditions: coeliac disease^45^, rheumatoid arthritis^43^, type 1 diabetes^46^, autoimmune hepatitis^47^ and also cardiovascular traits including coronary artery disease^48^ and blood pressure^49^. The genotype at rs653178 has been linked to levels of *SH2B3* mRNA expression in peripheral blood cell eQTL analysis (p = 9.24 × 10^−12^), although this association is not present in public eQTL data sets. Interestingly, rs3184504 T is generally the risk allele in autoimmune traits, suggesting opposing effects of the functional polymorphism on cancer and other traits, perhaps *via* shared effects on immune activation. A similar phenomenon has been found for the *HNF1B* SNP rs4430796 which has opposing effects on EC and type 2 diabetes risk^50^.

The *TERT*-*CLPTM1L* locus has been identified in multiple cancer susceptibility GWAS^51^,^52^,^53^,^54^,^55^,^56^,^57^,^58^ and it is of interest that the CRC SNP rs2736100 also shows signs of significance in EC in our analysis (OR:1.08, 95%CI:1.04-1.12, P = 1.67 × 10^−4^). In parallel with this study and using overlapping data sets, we have recently performed a detailed analysis of the *TERT-CLPTM1L* locus in EC which provided evidence that rs7705526 is associated with EC risk (P_assoc_ = 7.7 × 10^−5^), albeit at locus-specific rather than genome-wide significance thresholds^35^. rs7705526 is moderately correlated with rs2736100 (r^2^ ~ 0.5) but is poorly tagged in most Illumina GWAS arrays. Supplementary Figure 2 shows the complex LD structure between these two SNPs and 4 other SNPs previously associated with CRC and EC at varying levels of significance (P = 8.4 × 10^−3^ to 4.9 × 10^−6^) at this locus.

The rs2736100 A allele is the risk allele for CRC and testicular germ cell tumour, while the same allele is protective for EC, glioma and lung cancer, suggesting that this variant has its effects in a tissue-specific manner. Interestingly, we have found evidence in this study for a SNP (rs12970291, chromosome 18q22) that has opposing allelic effects on CRC and EC risk. The top candidate gene in this region is *TSHZ1* which encodes zinc finger homeodomain factor teashirt zinc finger family member 1, a protein involved in skin, skeletal, brain and gut development^59^ that is functionally related to the CRC gene *BMP4*^60^. One of several candidate SNPs near and within *TSHZ1* is the uncommon missense variant rs33930274 (p.Ala468Thr) in the last exon of *TSHZ1*, although the predicted functional consequences of this change are inconsistent (Grantham score = 58, SIFT = 0.0, Polyphen2 = 0.0, CADD score PHRED-scaled: 0.001).

Apart from the *SH2B3* and *TERT* SNPs, only two of 27 previously-reported CRC SNPs, including one near *TERC*, showed any good evidence of association with EC and neither of the known EC SNPs was associated with CRC risk. Otherwise, there was no convincing evidence for a shared EC and CRC predisposition based on common polymorphisms, although it will be important to keep repeating multi-cancer GWAS as more risk SNPs are identified, and sub-set analyses – for example of MSI+ ECs and CRCs – might also be fruitful. It remains a little puzzling that, like breast and ovarian cancer, CRC and EC share high-penetrance risk alleles, yet relatively few common risk alleles of modest effect.

## Additional Information

**How to cite this article**: Cheng, T. H.T. *et al.* Meta-analysis of genome-wide association studies identifies common susceptibility polymorphisms for colorectal and endometrial cancer near *SH2B3* and *TSHZ1*. *Sci. Rep.* **5**, 17369; doi: 10.1038/srep17369 (2015).

## Supplementary Material

Supplementary Information

## Figures and Tables

**Table 1:** Details of the CRC and EC studies used in this analysis.

		Study	Case sampling frame	Control sampling fram	Genotyping Platform	Cases	Controls
	CRC GWAS						
1	UK1-CORGI	Colorectal Tumour Gene Identification Consortium	England; Genetics clinic-based, with family history of CRC	England; spouses and partners of cases with no personal or family history of colorectal neoplasia	Illumina Hap550	888	899
2	Scotland1	Scotland	Scotland; population based CRC cases, age <55	Scotland; from NHS registers matched by age and region	Illumina HumanHap300 and Illumina HumanHap240S	973	998
3	VQ	VICTOR/QUASAR2	UK; CRC cases enrolled in chemotherapy clinical trials (NSAID and monoclonal antibody)		Illumina HumanHap300, Illumina HumanHap270, Illumina Human 1.2MDuo	1894	2674
	WTCCC2 BC58	UK 1958 Birth Cohort		UK; population based controls, born within one week in 1958	Illumina 1.2M		
4	CFR1	Colon Cancer Family Registry Phase1	USA and Australia; cases from cancer registries	USA and Australia; population based controls, no family history	Illumina Human1M	1175	999
5	CFR2	Colon Cancer Family Registry Phase 2	USA and Australia; cases from cancer registries		Illumina Human1M	795	
	CGEMS prostate	Cancer Genetic Markers of Susceptibility (Prostate)		USA; population based cancer free controls from prostate study	Illumina HumanHap550		1101
	EC GWAS						
6	NSECG	National Study of Endometrial Cancer Genetics	UK; population based cases		Illumina660WQuads, HumanHap550	925	
	CGEMS breast	Cancer Genetic Markers of Susceptibility (Breast)		USA; population based cancer free controls from breast study	Illumina HumanHap550		1141
7	ANECS	Australian National Endometrial Cancer Study	Australia; population based cases		Illumina 610K	606	
	QIMR	Queensland Institute of Medical Research		Australia; parents of participants in adolescent twin study	Illumina 610K		1846
	HCS	Hunter Community Study		Australia; population-based cohort	Illumina 610K		1237
8	SEARCH	UK Studies of Epidemiology and Risk factors in Cancer Heredity	England; population based cases via cancer registries, age <69		Illumina 610K	681	
	WTCCC2 NBS			UK; population based controls identified through National Blood Service	Illumina 1.2M		2501
	EC COGS						
9	ANECS	Australian National Endometrial Cancer Study	Australia; population based cases		Illumina Infinium iSelect	373	
	NECS	Newcastle Endometrial Cancer Study	Australia; hospital-based cases		Illumina Infinium iSelect	165	
	ABCFS	Australian Breast Cancer Family Study		Australia; from electoral rolls	Illumina Infinium iSelect		443
	AOCS	Australian Ovarian Cancer Study		Australia; population-based, from electoral rolls	Illumina Infinium iSelect		817
	MCCS	Melbourne Collaborative Cohort Study		Australia; random sample from initial cohort	Illumina Infinium iSelect		437
10	SEARCH	UK Studies of Epidemiology and Risk factors in Cancer Heredity	England; population based cases	England; population based controls	Illumina Infinium iSelect	773	7,510
11	NSECG	National Study of Endometrial Cancer Genetics	England; population based cases		Illumina Infinium iSelect	965	
	BBCS	British Breast Cancer Study		UK; friend, sister-in-law, daughter-in-law or other non-blood relative of breast cancer case	Illumina Infinium iSelect		1,353
	SBCS	Sheffield Breast Cancer Study		UK; women attending Sheffield Mammography Screening, with no breast lesion	Illumina Infinium iSelect		835
	UKBGS	UK Breakthrough Generations Study		UK; women without breast lesions selected from BGS cohort	Illumina Infinium iSelect		449
12	MECS	Mayo Endometrial Cancer Study	USA; Hospital based cases		Illumina Infinium iSelect	221	
	MCBCS	Mayo Clinic Breast Cancer Study		USA; Cancer-free women presenting for general medical examination	Illumina Infinium iSelect		1,762
	MCBCS/MCOCCCS	Mayo Clinic Ovarian Cancer Case-Control Study		USA; Cancer-free women presenting for general medical examination	Illumina Infinium iSelect		593
13	LES	Leuven Endometrial Cancer Study	Belgium; hospital based cases		Illumina Infinium iSelect	321	
	LMBC	Leuven Multidisciplinary Breast Centre		Belgium; controls from blood donors	Illumina Infinium iSelect		1,382
14	BECS/HJECS	Bavarian/Hannover-Jena Endometrial Cancer Study	Germany; population and hospital-based cases		Illumina Infinium iSelect	137	
	BBCC	Bavarian Breast Cancer Cases and Controls		Germany; healthy women >55yrs from newspaper advertisement	Illumina Infinium iSelect		441
	BSUCH	Breast Cancer Study of the University Clinic Heidelberg		Germany; female blood donors	Illumina Infinium iSelect		920
	ESTHER	ESTHER Breast Cancer Study		Germany; random sample from routine health check-up	Illumina Infinium iSelect		486
	GC-HBOC	German Consortium for Hereditary Breast & Ovarian Cancer		Germany; KORA study	Illumina Infinium iSelect		138
	GENICA	Gene Environment Interaction and Breast Cancer in Germany		Germany; random address sample	Illumina Infinium iSelect		420
	MARIE	Mammary Carcinoma Risk Factor Investigation		Germany; randomly drawn from population registries	Illumina Infinium iSelect		1,712
15	MoMaTEC	Molecular Markers in Treatment of Endometrial Cancer	Norway; population based cases		Illumina Infinium iSelect	599	
	NBCS	Norwegian Breast Cancer Study		Norway; attendees at Norwegian Breast Cancer Screening Program	Illumina Infinium iSelect		234
16	CAHRES/RENDOCAS	Cancer Hormone Replacement Epidemiology	Sweden; population based cases		Illumina Infinium iSelect	543	
	RENDOCAS	Registry of Endometrial Cancer in Sweden	Sweden; hospital based cases		Illumina Infinium iSelect	233	
	KARBAC	Karolinska Breast Cancer Study		Sweden; blood donors	Illumina Infinium iSelect		6,917
	pKARMA	Karolinska Mammography Project for Risk Prediction of Breast Cancer		Sweden; cancer-free participants of mammography screening	Illumina Infinium iSelect		6,917

**Table 2:** Association statistics for the known CRC SNPs tested in EC, and vice versa.

Cancer GWAS	SNP	Chr	Position (build 37)	Nearby gene(s)	Minor Allele	MAF	P-value in other phenotype	OR (minor allele)	L95 CI	U95 CI	Same effect direction in CRC and EC?	iCOGS EC samples included?	Reference
CRC	rs1801133	1	11,856,378	MTHFR	A	0.34	0.686	0.99	0.92	1.06	Yes	No	Hubner *et al.* Int Journal Cancer2006
CRC	rs10911251	1	183,081,194	LAMC1	C	0.43	0.236	1.04	0.97	1.12	No	No	Peters *et al.* Gastroenterology 2013, Whiffin *et al.* Hum Mol Genet 2014
CRC	rs6691170	1	222,045,446	DUSP10	T	0.37	0.023	1.09	1.01	1.17	Yes	No	Houlston *et al.* Nat Gen 2010
CRC	rs10936599	3	169,492,101	TERC	T	0.24	0.033	0.92	0.84	0.99	Yes	No	Houlston *et al.* Nat Gen 2010
CRC	rs2736100	5	1,286,516	TERT	A	0.5	**0.000167**	0.93	0.89	0.96	No	Yes	Kinnersley Br J Cancer 2012, Rafnar *et al.* Nat Gen 2009 Peters *et al.* Human Genetics 2012
CRC	rs647161	5	134,499,092	PITX1	C	0.33	0.559	1.02	0.95	1.1	No	No	Jia *et al.* Nat Gen 2013, Whiffin *et al.* Hum Mol Genet 2014
CRC	rs1321311	6	36,622,900	CDKN1A	A	0.24	0.925	1.00	0.92	1.08	No	No	Dunlop *et al.* Nat Gen 2012
CRC	rs16892766	8	117,630,683	EIF3H	C	0.09	0.134	0.95	0.88	1.02	No	Yes	Tomlinson *et al.* Nat Gen 2008
CRC	rs6983267	8	128,413,305	MYC	T	0.46	0.143	1.03	0.99	1.07	No	Yes	Tomlinson *et al.* Nat Gen 2007
CRC	rs10795668	10	8,701,219	GATA3	A	0.32	0.715	0.99	0.92	1.06	Yes	No	Tomlinson *et al.* Nat Gen 2008
CRC	rs1035209	10	101,345,366	NKX2-3, SLC25A28	T	0.2	0.243	1.05	0.97	1.15	Yes	No	Whiffin *et al.* Hum Mol Genet 2014
CRC	rs3824999	11	74,345,550	POLD3	T	0.49	0.647	0.98	0.92	1.05	Yes	No	Dunlop *et al.* Nat Gen 2012
CRC	rs3802842	11	111,171,709	COLCA1, COLCA2, POU2AF1	C	0.31	0.513	0.99	0.94	1.03	No	Yes	Tenesa *et al.* Nat Gen 2008
CRC	rs10774214	12	4,368,352	CCND2	T	0.38	0.171	1.05	0.98	1.13	Yes	Yes	Jia *et al.* Nat Gen 2013, Whiffin *et al.* Hum Mol Genet 2014
CRC	rs3217810	12	4,388,271	CCND2	T	0.14	0.762	1.02	0.92	1.13	Yes	No	Peters *et al.* Gastroenterology 2013, Whiffin *et al.* Hum Mol Genet 2014
CRC	rs11169552	12	51,155,663	DIP2B, ATF1	T	0.26	0.963	1.00	0.93	1.08	No	No	Houlston *et al.* Nat Gen 2010
CRC	rs4444235	14	54,410,919	BMP4	C	0.48	0.1	1.03	0.99	1.07	Yes	Yes	Houlston *et al.* Nat Gen 2008
CRC	rs1957636	14	54,560,018	BMP4	T	0.41	0.961	1.00	0.96	1.04	No	Yes	Tomlinson *et al.* PLoS Genetics 2011
CRC	rs16969681	15	32,993,111	GREM1	T	0.09	0.379	0.97	0.90	1.04	No	Yes	Tomlinson *et al.* PLoS Genetics 2011
CRC	rs11632715	15	33,004,247	GREM1	A	0.48	0.332	1.04	0.97	1.11	Yes	No	Tomlinson *et al.* PLoS Genetics 2011
CRC	rs9929218	16	68,820,946	CDH1, CDH3	A	0.29	0.679	0.98	0.91	1.06	Yes	No	Houlston *et al.* Nat Gen 2008
CRC	rs4939827	18	46,453,463	SMAD7	C	0.46	0.229	0.98	0.94	1.02	Yes	Yes	Broderick *et al.* Nat Gen 2007
CRC	rs10411210	19	33,532,300	RHPN2	T	0.09	0.202	1.04	0.98	1.12	No	Yes	Houlston *et al.* Nat Gen 2008
CRC	rs961253	20	6,404,281	BMP2	A	0.37	0.975	1.00	0.96	1.04	No	Yes	Houlston *et al.* Nat Gen 2008
CRC	rs4813802	20	6,699,595	BMP2	G	0.37	0.268	1.04	0.97	1.12	Yes	No	Tomlinson *et al.* PLoS Genetics 2011
CRC	rs2423279	20	7,812,350	HAO1	C	0.24	0.897	1.01	0.93	1.09	Yes	No	Jia *et al.* Nat Gen 2013, Whiffin *et al.* Hum Mol Genet 2014
CRC	rs4925386	20	60,921,044	LAMA5	T	0.3	0.064	1.07	1.00	1.16	No	No	Houlston *et al.* Nat Gen 2010, Peters *et al.* Human Genetics 2012
EC	rs749292*	15	51,558,731	CYP19A1	A	0.46	0.066	0.95	0.91	1.00	No	Yes	Spurdle *et al.* Nat Gen 2011
EC	rs4430796*	17	36,098,040	HNF1B	G	0.47	0.601	0.99	0.94	1.04	Yes	Yes	Setiawan *et al.* Cancer Epidemiol Biomarkers Prev 2009

Chr = chromosome, OR = odds ratio, MAF = minor allele frequency, OR = odds ratio, L95 CI = lower 95% confidence interval odds ratio, U95 CI = upper 95% confidence interval odds ratio. The original studies providing the data are listed in Supplementary Information.
