# Supplementary material for: Meta-analysis of genome-wide association studies identifies common susceptibility polymorphisms for colorectal and endometrial cancer near SH2B3 and TSHZ1
Source: Sci Rep. 2015 Dec 1;5:17369. doi: 10.1038/srep17369 (PMC4664893; doi:10.1038/srep17369)
Supplement: Supplementary Information [file srep17369-s1.pdf]

Supplementary Information

Meta-analysis of genome-wide association studies identifies common susceptibility polymorphisms for colorectal and endometrial cancer near *SH2B3* and *TSHZ1*

Timothy HT Cheng[1], Deborah Thompson[2], Jodie Painter[3], Tracy O'Mara[3], Maggie Gorman[1], Lynn Martin[1], Claire Palles[1], Angela Jones[1], Daniel D. Buchanan[4,5], Aung Ko Win[5], John Hopper[5], Mark Jenkins[5], Noralane M. Lindor[6], Polly A. Newcomb[7], Steve Gallinger[8], David Conti[9], Fred Schumacher[9], Graham Casey[9], Graham G Giles[5,10,11], Paul Pharoah[2,12], Julian Peto[13], Angela Cox[14], Anthony Swerdlow[15], Fergus Couch[16,17], Julie M Cunningham[16], Ellen L Goode[17], Stacey J Winham[17], Diether Lambrechts[18], Peter Fasching[19,20], Barbara Burwinkel[21,22], Hermann Brenner[22,23], Hiltrud Brauch[22,23], Jenny Chang-Claude[23], Helga B. Salvesen[24], Vessela Kristensen[25], Hatef Darabi[26], Jingmei Li[26], Tao Liu[26], Annika Lindblom[26], Per Hall[27], Magdalena Echeverry de Polanco[28], Monica Sans[29], Angel Carracedo[30], Sergi Castellvi-Bel[31], Augusto Rojas-Martinez[32], Samuel Aguiar Jnr[33], Manuel R. Teixeira[34], Alison M Dunning[12], Joe Dennis[2], Geoffrey Otton[35], Tony Proietto[35], Elizabeth Holliday[36], John Attia[36], Katie Ashton[36], Rodney J Scott[36], Mark McEvoy[37], Sean C Dowdy[38], Brooke L Fridley[39], Henrica MJ Werner[40], Jone Trovik[40], Tormund S Njolstad[40], Emma Tham[26], Miriam Mints[41], Ingo Runnebaum[42], Peter Hillemanns[43], Thilo Dörk[44], Frederic Amant[45], Stefanie Schrauwen[18], Alexander Hein[20], Matthias W Beckmann[20], Arif Ekici[46], Kamila Czene[27], Alfons Meindl[47], Manjeet K Bolla[2], Kyriaki Michailidou[2], Jonathan P Tyrer[12], Qin Wang[2], Shahana Ahmed[12], Catherine S Healey[12], Mitul Shah[12], Daniela Annibali[48], Jeroen Depreeuw[48], Australian Ovarian Cancer Study Group[$], Nada A. Al-Tassan[50], Rebecca Harris[51], Brian F. Meyer[50], Nicola Whiffin[52], Fay J Hosking[52], Ben Kinnersley[52], Susan M. Farrington[53], Maria Timofeeva[53], Albert Tenesa[54], Harry Campbell[55], Robert W. Haile[56], Shirley Hodgson[57], Luis Carvajal-Carmona[58], Jeremy P. Cheadle[51], Douglas Easton[2,12], Malcolm Dunlop[53], Richard Houlston[52], Amanda Spurdle[3], Ian Tomlinson[1,59]

[1] Molecular and Population Genetics Laboratory, Wellcome Trust Centre for Human Genetics, University of Oxford, Roosevelt Drive, Oxford OX3 7BN, UK

[2] Centre for Cancer Genetic Epidemiology, Public Health and Primary Care, University of Cambridge, Cambridge CB1 8RN, UK

[3] The Molecular Cancer Epidemiology Laboratory, QIMR Berghofer Medical Research Institute, Brisbane 4006, Australia

[4] Oncogenomics Group, Genetic Epidemiology Laboratory, Department of Pathology, The University of Melbourne, Victoria, Australia

[5] Centre for Epidemiology and Biostatistics, The University of Melbourne, Victoria, Australia

[6] Department of Health Sciences Research, Mayo Clinic, Scottsdale, AZ, USA

[7] Cancer Prevention Program, Fred Hutchinson Cancer Research Center, Seattle, WA, USA
[8] Lunenfeld-Tanenbaum Research Institute, Mount Sinai Hospital, Toronto, ON, Canada
[9] Department of Preventive Medicine, University of Southern California, Los Angeles, CA, USA
[10] Cancer Epidemiology Centre, Cancer Council Victoria, Melbourne, Australia
[11] Department of Epidemiology and Preventive Medicine, Monash University, Melbourne, Australia
[12] Centre for Cancer Genetic Epidemiology, Department of Oncology, University of Cambridge, Cambridge, UK
[13] London School of Hygiene and Tropical Medicine, London, UK
[14] Sheffield Cancer Research Centre, Department of Oncology, University of Sheffield, Sheffield, UK
[15] Division of Genetics and Epidemiology, Institute of Cancer Research, Sutton, UK and 17 Division of Breast Cancer Research, Institute of Cancer Research, London, UK
[16] Department of Laboratory Medicine and Pathology, Mayo Clinic, Rochester, MN, USA
[17] Department of Health Sciences Research, Mayo Clinic, Rochester, MN, USA.
[18] Department of Oncology, KU Leuven, Belgium
[19] University of California at Los Angeles, Department of Medicine, Division of Hematology/Oncology, David Geffen School of Medicine, Los Angeles, CA, USA
[20] Department of Gynecology and Obstetrics, University Hospital Erlangen, Friedrich-Alexander University Erlangen-Nuremberg, Erlangen, Germany
[21] Division of Cancer Epidemiology, German Cancer Research Center, Heidelberg, Germany
[22] Dr. Margarete Fischer-Bosch-Institute of Clinical Pharmacology Stuttgart, University of Tuebingen, Germany
[23] Division of Cancer Epidemiology, German Cancer Research Center, Heidelberg, Germany
[24] Department of Clinical Science, Center for Cancer Biomarkers, University of Bergen, Norway
[25] Department of Genetics, Institute for Cancer Research, The Norwegian Radium Hospital, Oslo, Norway; The K.G. Jebsen Center for Breast Cancer Research, Institute for Clinical Medicine, Faculty of Medicine, University of Oslo, Oslo, Norway; Department of Clinical Molecular Oncology, Division of Medicine, Akershus University Hospital, Ahus, Norway
[26] Department of Molecular Medicine and Surgery, Karolinska Institutet, Stockholm, Sweden
[27] Department of Medical Epidemiology and Biostatistics, Karolinska Institutet, Stockholm, Sweden
[28] Grupo de investigación Citogenética, Filogenia y Evolución de Poblaciones, Universidad del Tolima, Ibagué, Tolima, Colombia
[29] Departamento de Antropologia Biologica, Facultad de Humanidades, UDELAR, Magallanes 1577, CP 11200, Montevideo, Uruguay
[30] Universidade de Santiago de Compostel,R/ San Francisco s/n 15782, Santiago de Compostela, Spain

[31] Genetic Predisposition to Colorectal Cancer Group, Gastrointestinal & Pancreatic Oncology Team, IDIBAPS/CIBERehd/Hospital Clínic, Centre Esther Koplowitz (CEK), Rosselló 153 planta 4, 08036 Barcelona, Spain
[32] Universidad Autónoma De Nuevo León, Pedro de Alba s/n, San Nicolás de Los Garza, Nuevo León, Mexico
[33] Hospital A.C. Camargo, São Paulo, Brazil
[34] Department of Genetics and IPO-Porto Research Center (CI-IPOP), Portuguese Oncology Institute of Porto (IPO-Porto), Porto, Portugal, and Biomedical Sciences Institute (ICBAS), University of Porto, Porto, Portugal
[35] School of Medicine and Public Health, University of Newcastle, NSW, Australia
[36] Hunter Medical Research Institute, John Hunter Hospital, Newcastle, NSW, Australia
[37] Centre for Clinical Epidemiology and Biostatistics, School of Medicine and Public Health, University of Newcastle, NSW, Australia
[38] Department of Obstetrics and Gynecology, Division of Gynecologic Oncology, Mayo Clinic, Rochester, MN, USA
[39] Department of Biostatistics, University of Kansas Medical Center, Kansas City, KS, USA
[40] Centre for Cancer Biomarkers, Department of Clinical Science, The University of Bergen, Norway
[41] Department of Women's and Children's Health, Karolinska Institutet, Karolinska University Hospital, Stockholm, Sweden
[42] Department of Gynaecology, Jena University Hospital - Friedrich Schiller University, Jena, Germany
[43] Hannover Medical School, Clinics of Gynaecology and Obstetrics, Hannover, Germany
[44] Hannover Medical School, Gynaecology Research Unit, Hannover, Germany
[45] Division of Gynaecological Oncology, University Hospital Leuven, Leuven, Belgium
[46] Institute of Human Genetics, University Hospital Erlangen, Friedrich-Alexander University Erlangen-Nuremberg, Erlangen, Germany
[47] Department of Obstetrics and Gynecology, Division of Tumor Genetics, Technical University of Munich, Munich, Germany
[48] Department of Obstetrics and Gynecology, Division of Gynecologic Oncology, University Hospitals, KU Leuven, University of Leuven, 3000, Belgium
[49] Vesalius Research Center, Leuven, 3000, Belgium
[50] Department of Genetics, King Faisal Specialist Hospital and Research Center, P.O.Box 3354, Riyadh11211, Saudi Arabia
[51] Institute of Cancer and Genetics, School of Medicine, Cardiff University, Heath Park, Cardiff, CF14 4XN, UK

[52] Division of Genetics and Epidemiology, The Institute of Cancer Research, Sutton, Surrey SM2 5NG, UK
[53] Colon Cancer Genetics Group, Institute of Genetics and Molecular Medicine, University of Edinburgh and MRC Human Genetics Unit, Western General Hospital Edinburgh, Crewe Road, Edinburgh, EH4 2XU, UK
[54] The Roslin Institute, University of Edinburgh, Easter Bush, Roslin, EH25 9RG, UK
[55] Centre for Population Health Sciences, University of Edinburgh, Edinburgh, EH8 9AG, UK
[56] Stanford Cancer Institute, Lorry Lokey Building/SIM 1, 265 Campus Drive, Ste G2103, Stanford, CA 94305-5456, USA.
[57] Department of Cancer Genetics, St. George's University of London, London SW17 0RE, UK
[58] Genome Center and Department of Biochemistry and Molecular Medicine, School of Medicine, University of California, Davis, USA
[59] Oxford NIHR Comprehensive Biomedical Research Centre, Wellcome Trust Centre for Human Genetics, Roosevelt Drive, Oxford OX3 7BN, UK.

[$] A list of participants and their affiliations appears in the Supplementary Information.

[+] to whom correspondence should be addressed at Wellcome Trust Centre for Human Genetics, Roosevelt drive, Oxford OX3 7BN, United Kingdom. Email iant@well.ox.ac.uk

Consortium

The Australian Ovarian Cancer Study (AOCS) group.
Management Group: D Bowtell (Peter MacCallum Cancer Centre), G Chenevix-Trench, A Green, P Webb (QIMR), A deFazio (Westmead Institute for Cancer Research, WMI), D Gertig (Victorian Cervical Cytology Registry)
Project Managers: N Traficante, S Fereday, K Alsop (Peter MacCallum Cancer Centre), S Moore (QIMR), J Hung (Westmead Institute for Cancer Research, WMI)
Data Managers: S Fereday (Peter MacCallum Cancer Centre), K Harrap, T Sadkowsky, N Pandeya (QIMR)
Research Nurses and Assistants: ACT- M Malt; NSW- A Mellon, R Robertson (John Hunter Hospital), T Vanden Bergh, M Jones, P Mackenzie (Royal Hospital for Women), J Maidens (Royal North Shore Hospital), K Nattress (Royal Prince Alfred Hospital), YE Chiew, A Stenlake, H Sullivan, (Westmead Hospital); QLD- B Alexander, P Ashover, S Brown, T Corrish, L Green, L Jackman, K Ferguson, K Martin, A Martyn, B Ranieri (QIMR); SA- J White (Flinders Medical Centre, Royal Adelaide Hospital, Queen Elizabeth); TAS- V Jayde (Royal Hobart Hospital); VIC- L Bowes (Freemasons Hospital, Mercy Hospital for Women, Royal Women's Hospital); P Mamers (Monash Medical Centre); L Galletta, D Giles, J Hendley, K Alsop (Peter MacCallum Cancer Centre); WA- T Schmidt, H Shirley, C Ball, C Young, S Viduka, Hoa Tran, Sanela Bilic, Lydia Glavinas (WARTN), Julia Brooks (St John of God Hospital)
Clinical Collaborators: ACT- R Stuart-Harris (Canberra Hospital); NSW- F Kirsten (Bankstown); J Rutovitz (Hornsby); P Clingan, A Glasgow (Illawarra Area Health Services); A Proietto, S Braye, G Otton (John Hunter Hospital); J Shannon (Nepean Hospital); T Bonaventura, J Stewart (Newcastle Mater Misericordiae); S Begbie (Port Macquarie Base Hospital); M Friedlander (Prince of Wales Hospital); D Bell, S Baron-Hay, A Ferrier (dec.), G Gard, D Nevell, N Pavlakis, S Valmadre, B Young (until mid 2003) (Royal North Shore Hospital); C Camaris, R Crouch, L Edwards, N Hacker, D Marsden, G Robertson (Royal Hospital for Women); P Beale, J Beith, J Carter, C Dalrymple, R Houghton, P Russell, L Anderson (Royal Prince Alfred Hospital); M Links (St George Hospital); J Grygiel (St Vincent's Hospital); J Hill (Wagga Wagga Base Hospital); A Brand, K Byth, R Jaworski, P Harnett, R Sharma, G Wain (Westmead Hospital); QLD- B Ward, D Papadimos (Mater Misericordiae Hospitals); A Crandon, M Cummings, K Horwood. A Obermair, L Perrin, D Wyld (Royal Women's Hospital); J Nicklin (Royal Women's Hospital and Wesley Hospital); SA- M Davy, MK Oehler, C Hall, T Dodd, T Healy, K Pittman (Royal Adelaide Hospital, Burnside Memorial Hospital); D Henderson (Flinders Medical Centre); J Miller, J Pierdes (Queen Elizabeth Hospital); A Achan (ICPMR); TAS- P Blomfield, D Challis, R McIntosh, A Parker (Royal Hobart Hospital); VIC- B Brown, R Rome (Freemasons Hospital); D Allen, P Grant, S Hyde, R Laurie M Robbie, (Mercy

Hospital for Women), D Healy, T Jobling, T Manolitsas, J McNealage, P Rogers, B Susil, E Sumithran, I Simpson, (Monash Medical Centre); K Phillips, D Rischin, S Fox, D Johnson, P Waring, S Lade, M Loughrey, N O'Callaghan, W Murray, L Mileshkin, P Allan (Peter MacCallum Cancer Centre); V Billson, J Pyman, D Neesham, M Quinn, A Hamilton, O McNally (Royal Women's Hospital); C Underhill (Border Medical Oncology); Prof R Bell (Andrew Love Cancer Centre); LF Ng (Ballarat Base Hospital); R Blum (Bendigo Health Care Group); V Ganju (Peninsula Health); WA- I Hammond, Y Leung (School for Women's and Infants' Health, University of Western Australia and the Western Australian Gynaecologic Cancer Service); A McCartney (dec.), C Stewart (King Edward Memorial Hospital); M Buck (Mount Hospital)
Scientific Collaborators: I Haviv (Peter MacCallum Cancer Centre); D Purdie, D Whiteman (QIMR); N Zeps (WARTN)
Scientific Advisory Board: P Gurry, A Hamilton, S Hankinson, P Meltzer

SUPPLEMENTARY DATA

Supplementary Information. Primary data sources for the 29 previously-reported CRC and EC SNPs.

Broderick et al. A genome-wide association study shows that common alleles of SMAD7 influence colorectal cancer risk. Nat Genet. 2007;39:1315–1317.

Dunlop et al. Common variation near CDKN1A, POLD3 and SHROOM2 influences colorectal cancer risk. Nat Genet. 2012 May 27;44(7):770-6.

Houlston et al. Meta-analysis of genome-wide association data identifies four new susceptibility loci for colorectal cancer. Nat Genet. 2008 Dec;40(12):1426-35.

Houlston et al. Meta-analysis of three genome-wide association studies identifies susceptibility loci for colorectal cancer at 1q41, 3q26.2, 12q13.13 and 20q13.33. Nat Genet. 2010 Nov;42(11):973-7.

Jia et al. Genome-wide association analyses in east Asians identify new susceptibility loci for colorectal cancer. Nature Genetics 45, 191–196 (2013)

Kinnersley et al. The TERT variant rs2736100 is associated with colorectal cancer risk. Br J Cancer. 2012 Sep 4;107(6):1001-8.

Peters et al. Identification of genetic susceptibility loci for colorectal tumors in a genome-wide meta-analysis. Gastroenterology. 2013 Apr;144(4):799-807.e24.

Peters et al. Meta-analysis of new genome-wide association studies of colorectal cancer risk. Hum Genet. 2012 Feb;131(2):217-34.

Rafnar et al. Sequence variants at the TERT-CLPTM1L locus associate with many cancer types. Nat Genet. 2009 Feb;41(2):221-7.

Setiawan et al. Two estrogen-related variants in CYP19A1 and endometrial cancer risk: a pooled analysis in the Epidemiology of Endometrial Cancer Consortium. 2009 Jan;18(1):242-7.

Spurdle et al. Genome-wide association study identifies a common variant associated with risk of endometrial cancer. Nat Genet. 2011 May;43(5):451-4.

Tenesa et al. Genome-wide association scan identifies a colorectal cancer susceptibility locus on 11q23 and replicates risk loci at 8q24 and 18q21. Nat Genet. 2008 May;40(5):631-7.

Tomlinson et al. A genome-wide association scan of tag SNPs identifies a susceptibility variant for colorectal cancer at 8q24.21. Nat Genet. 2007 Aug;39(8):984-8.

Tomlinson et al. A genome-wide association study identifies colorectal cancer susceptibility loci on chromosomes 10p14 and 8q23.3. Nat Genet. 2008;40:623–630.

Tomlinson et al. Multiple common susceptibility variants near BMP pathway loci GREM1, BMP4, and BMP2 explain part of the missing heritability of colorectal cancer. PLoS Genet. 2011 Jun;7(6):e1002105.

Whiffin et al. Identification of susceptibility loci for colorectal cancer in a genome-wide meta-analysis. Hum Mol Genet. 2014 Sep 1;23(17):4729-37.
Zhang et al. Large-scale genetic study in East Asians identifies six new loci associated with colorectal cancer risk. Nature Genetics 46, 533–542 (2014)

Supplementary Table 1. Association statistics of SNPs previously undiscovered by GWAS with strongest evidence of effects on combined CRC and EC predisposition.

SNP	Chr	Position (build 37)	Nearby gene	Reference allele	Allele frequency	OR	L95 CI	U95 CI	p-value	I^2	iCOGS EC samples included?
**rs3184504**	**12**	**111,884,608**	**SH2B3**	**C**	**0.520**	**1.10**	**1.06**	**1.13**	**8.23E-09**	**0.00**	**Yes**
rs6051080	20	25,975,674	NANP	A	0.649	1.12	1.08	1.17	1.23E-07	0.00	No
rs2052678	12	29,833,329	TMTC1	G	0.861	0.86	0.81	0.91	1.38E-07	0.31	No
rs11085466	19	21,751,811	ZNF429	G	0.782	1.15	1.09	1.22	1.53E-07	0.00	No
rs17503919	6	89,565,737	RNGTT	A	0.862	1.17	1.11	1.25	1.66E-07	0.00	No
rs76225372	10	19,320,086	ARL5B	A	0.912	1.21	1.13	1.31	1.68E-07	0.00	No
rs7740797	6	155,121,259	SCAF8	G	0.513	1.12	1.07	1.16	2.20E-07	0.36	No
rs3181245	6	24,651,320	TDP2	C	0.514	1.12	1.07	1.16	2.23E-07	0.45	No
rs4378954	3	115,650,448	LSAMP	C	0.898	1.15	1.09	1.21	4.05E-07	0.10	Yes
rs10457678	6	139,122,240	ECT2L	A	0.773	1.10	1.06	1.14	4.12E-07	0.00	Yes
rs17035310	4	106,064,754	TET2	C	0.876	1.13	1.08	1.18	6.10E-07	0.00	Yes
rs9901225	17	40,755,811	PSMC3IP	T	0.551	1.11	1.06	1.16	1.13E-06	0.49	No
rs1512436	11	106,306,871	GUCY1A2	T	0.553	0.93	0.90	0.95	1.22E-06	0.00	Yes
rs10217586	9	22,121,349	CDKN2B-AS1	T	0.539	0.93	0.90	0.96	1.83E-06	0.74	Yes
rs12446552	16	11,720,066	LITAF	T	0.723	0.92	0.89	0.95	3.05E-06	0.24	Yes
rs4853036	2	70,059,824	GMCL1	G	0.736	0.92	0.89	0.95	6.43E-06	0.00	Yes
rs4789378	17	74,930,366	MGAT5B	C	0.648	0.93	0.90	0.96	8.85E-06	0.51	Yes
rs2200650	11	7,326,195	SYT9	G	0.635	1.08	1.04	1.11	1.19E-05	0.00	Yes

Supplementary Table 2. Functional annotation of the region around rs3184504.

SNP	Position on chr 12 (build 37)	LD (r^2) rs3184504	LD (D')	Referece allele	Allele Frequency (Europeans)	RefSeq genes	Location	GERP cons	SiPhy cons	Promoter histone marks	Enhancer histone marks	DNAse	Proteins	TCGA/Gtex eQTL	Motifs changed	RegulomeDB Score	Phast Cons	ENCODE RNA-seq RegTFBS-clustered	FunSeq ENCODE annotation
rs10774624	111,833,788	0.81	0.92	G	0.53	10kb 5' of SH2B3	intergenic								Ets,Nr2f2				
rs7310615	111,865,049	0.93	0.98	C	0.53	SH2B3	intronic				GM12878, Huvec				ERalpha-a				
**rs3184504**	**111,884,608**	**1**	**1**	**T**	**0.53**	**SH2B3**	**missense**				**Huvec, GM12878**				**HES1,Mtf1**	3a			
rs4766578	111,904,371	0.9	0.99	T	0.51	ATXN2	intronic				NHLF				ERalpha-a,GATA,GCNF,LRH1,RORalpha1,RXRA,SF1	6			
rs10774625	111,910,219	0.9	0.98	A	0.52	ATXN2	intronic				K562				Cdx,Foxf1,Foxi1,Foxj2,Foxl1,Foxo,Foxp1,Foxq1,Zfp105	5			
rs7137828	111,932,800	0.87	0.93	C	0.53	ATXN2	intronic								SP1	5			
rs597808	111,973,358	0.75	0.92	A	0.51	ATXN2	intronic								BCL,ERalpha-a,Ets,Irf,SP1,ZBRK1,Znf143				
rs653178	112,007,756	0.78	0.89	C	0.53	ATXN2	intronic			8 organs	NHLF, GM12878, HSMM, HMEC, K562, Huvec, NHEK				Esr2	5			Enhancer(2)
rs11065979	112,059,557	0.61	-0.83	C	0.44	20kb 3' of BRAP	intergenic								Myf	5			
rs11065987	112,072,424	0.51	-0.78	A	0.42	7.5kb 3' of BRAP	intergenic				K562	K562			Mrg1::Hoxa9	3a			DHS, Enhancer(2)
rs11065991	112,083,162	0.5	-0.77	C	0.42	BRAP	intronic								Myc	4			Enhancer, TFP(FOXA1, GATA3)

Gene-based location, distance from Refseq genes, LD, Phast cons, GERP, SiPhy scores from Haploreg and annovar. All SNPs with blank GERP scores map to a location that score <2 and so is regarded as not being evolutionarily conserved/potentially function al. All SNPs with blank Phastcons scores represent SNPs that do not map to within conserved regions. RegulomeDB score (http://regulomedb.org/) based on Known and predicted regulatory DNA elements from GEO, the ENCODE project, and published literature. Tissue-specific eQTL searches were made using Gtex. (http://www.gtexportal.org/home/) and TCGA data. FunSeq annotation usinghttp://funseq.gersteinlab.org.

Supplementary Table 3. Association statistics of SNPs previously undiscovered by CRC or EC GWAS with strongest evidence of effects in opposite directions on CRC and EC predisposition.

SNP	Chr	Position (build 37)	Nearby gene	Reference allele	Allele freq.	OR	L95 CI	U95 CI	p-value	I^2	iCOGS EC samples included?
rs2270539	1	78,047,628	ZZZ3	T	0.979	0.80	0.72	0.88	1.60E-05	0.00	Yes
rs34462569	2	166,552,918	CSRNP3	G	0.934	1.22	1.12	1.33	3.75E-06	0.03	No
rs10510663	3	35,559,240	ARPP21	T	0.948	0.86	0.80	0.92	2.28E-05	0.53	Yes
rs1735546	3	128,075,398	EEFSEC	T	0.752	1.08	1.04	1.12	3.11E-05	0.29	Yes
rs1852266	7	48,340,033	ABCA13	C	0.741	0.92	0.89	0.96	2.27E-05	0.00	Yes
rs4727012	7	148,740,493	EZH2	C	0.891	1.18	1.10	1.26	9.64E-07	0.34	No
rs112541862	9	97,483,158	FBP1	A	0.962	1.30	1.16	1.45	4.10E-06	0.03	No
rs77922938	9	111,060,196	KLF4	G	0.953	0.85	0.79	0.91	8.33E-06	0.00	Yes
rs606460	11	75,248,457	GDPD5	G	0.725	1.11	1.06	1.17	3.58E-06	0.29	No
rs11607499	11	81,811,229	PRCP	T	0.954	1.27	1.15	1.40	1.75E-06	0.00	No
rs997275	11	110,454,876	ARHGAP20	A	0.578	1.07	1.04	1.11	2.36E-05	0.00	Yes
rs12817211	12	50,579,398	LIMA1	C	0.767	1.12	1.07	1.18	2.76E-06	0.50	No
rs3117967	13	22,508,254	FGF9	G	0.896	0.89	0.85	0.94	1.33E-05	0.17	Yes
rs9592895	13	73,813,982	KLF5	T	0.655	1.08	1.04	1.11	1.42E-05	0.39	Yes
rs1952157	14	34,289,438	NPAS3	C	0.690	1.08	1.05	1.12	6.26E-06	0.52	Yes
rs3001371	14	105,242,831	AKT1	C	0.691	0.93	0.90	0.96	1.29E-05	0.30	Yes
rs11150038	16	78,076,559	CLEC3A	G	0.944	1.27	1.15	1.39	9.89E-07	0.00	No
rs76525880	17	46,614,250	HOXB1	A	0.929	1.16	1.09	1.24	6.68E-06	0.58	Yes
**rs12970291**	**18**	**73,011,336**	**TSHZ1**	**T**	**0.965**	**1.27**	**1.16**	**1.38**	**4.82E-08**	**0.00**	**Yes**
rs4816890	21	24,420,502	NCAM2	T	0.637	0.90	0.86	0.94	2.13E-06	0.24	No

Supplementary Table 4. Functional annotation of the region around rs12970291.

SNP	Position on chr 18 (build 37)	LD (r^2) rs12970291	LD (D')	Referece allele	Allele Frequency (Europeans)	RefSeq genes	Location	GERP cons	SiPhy cons	Promoter histone marks	Enhancer histone marks	DNAse	Proteins	TCGA/ Gtex eQTL	Motifs changed	RegulomeDB Score	Phast Cons	ENCODE RNA-seq RegTFBS-clustered	FunSeq ENCODE annotation
rs12969532	72,984,262	0.52	0.74	C	0.03	TSHZ1	intronic								Arnt,BHLHE40,GR	5			
rs71359079	72,984,455	0.5	0.71	C	0.03	TSHZ1	intronic								6 altered motifs	6			
rs12965321	72,986,176	0.59	0.83	G	0.02	TSHZ1	intronic								Hand1,Nanog,Sox,Zbtb12	5	Score 452; lod 38		
rs34981053	72,989,467	0.59	0.83	T	0.02	TSHZ1	intronic				Huvec, HSMM	35 cell types			HNF4,NRSF,SP1	5			DHS, TFP(ZNF263)
rs28420499	72,989,742	0.5	0.71	G	0.03	TSHZ1	intronic				Huvec, HSMM	5 cell types			7 altered motifs	5			DHS, TFP(ZNF263)
rs77026922	72,991,038	0.68	0.85	T	0.03	TSHZ1	intronic					7 cell types			13 altered motifs	5			DHS
rs56280961	72,991,193	0.4	0.78	C	0.02	TSHZ1	intronic					Medullo			Pax-5,Pax-6	5			DHS
rs34551261	72,991,577	0.68	0.85	G	0.03	TSHZ1	intronic					6 cell types	ZNF263		8 altered motifs	4		ZNF263	DHS, TFP(ZNF263)
rs36046989	72,996,430	0.73	0.85	C	0.03	TSHZ1	intronic					ProgFib			Foxa,Hsf,LXR,RXRA	4			
rs33930274	72,998,899	0.77	0.9	G	0.03	TSHZ1	missense					HMEC,GM12891,GM19239,Medullo			Pou2f2	5			
rs34242916	73,003,598	0.73	0.85	T	0.03	1.7kb 3' of TSHZ1	intergenic				HepG2	HepG2			NRSF	5			DHS
rs34704942	73,003,694	0.73	0.85	C	0.03	1.8kb 3' of TSHZ1	intergenic					LNCaP,Th1,ProgFib,iPS			5 altered motifs	5			DHS
rs12969489	73,008,360	0.81	0.95	C	0.03	6.5kb 3' of TSHZ1	intergenic								5 altered motifs				
rs9946654	73,011,336	0.81	0.95	T	0.03	9.4kb 3' of TSHZ1	intergenic								BRCA1,Foxp1,Nkx2				
rs36104458	73,012,489	0.81	0.95	A	0.03	11kb 3' of TSHZ1	intergenic					CLL			Gfi1	5			DHS
rs12962160	73,013,181	0.81	0.95	A	0.03	11kb 3' of TSHZ1	intergenic								HNF1,PLZF				
rs34227877	73,013,811	0.57	0.93	A	0.02	12kb 3' of TSHZ1	intergenic					Fibrobl			LBP-1,Pax-6,ZBTB7A	5			DHS
rs17056795	73,016,067	0.69	1	G	0.04	14kb 3' of TSHZ1	intergenic					Fibrobl			BCL,NF-kappaB,ZBTB33	5			DHS
**rs12970291**	73,017,234	**1**	**1**	**G**	**0.03**	**15kb 3' of TSHZ1**	**intergenic**								**9 altered motifs**	5			
rs12968532	73,020,946	1	1	C	0.03	19kb 3' of TSHZ1	intergenic								Hoxd10,LRH1,Mef2,Pax-1				
rs35969565	73,022,413	1	1	C	0.03	21kb 3' of TSHZ1	intergenic								GLI				
rs12959779	73,023,486	1	1	G	0.03	22kb 3' of TSHZ1	intergenic					Medullo,Osteobl			9 altered motifs	5			DHS
rs12965434	73,024,810	1	1	G	0.03	23kb 3' of TSHZ1	intergenic												
rs12961817	73,024,904	1	1	C	0.03	23kb 3' of TSHZ1	intergenic					Melano			EWSR1-FLI1,Ets,GATA,TATA	5			DHS
rs78784067	73,025,962	0.91	1	C	0.03	24kb 3' of TSHZ1	intergenic								Hmx,Sox	5			
rs2581665	73,030,498	1	-1	C	0.97	29kb 3' of TSHZ1	intergenic								BCL,NRSF,SP1	5			
rs12955930	73,030,682	1	1	C	0.03	29kb 3' of TSHZ1	intergenic								HDAC2,Pax-6,Pax-8	5			
rs35185115	73,032,159	1	1	A	0.03	30kb 3' of TSHZ1	intergenic					PanIslets			Foxp3,Mef2	5			TFP(PAX5)
rs34521731	73,032,447	0.91	1	TC	0.03	31kb 3' of TSHZ1	intergenic					Th1,FibroP,Medullo			11 altered motifs				
rs12969979	73,032,676	1	1	G	0.03	31kb 3' of TSHZ1	intergenic					LNCaP			YY1	5			
rs12966313	73,032,803	1	1	C	0.03	31kb 3' of TSHZ1	intergenic								Nrf-2,TCF11::MafG,ZID	6			
rs12956172	73,033,299	1	1	A	0.03	31kb 3' of TSHZ1	intergenic					LNCaP,Fibrobl,Osteobl			Ik-1,Ik-2,ZEB1	2b	Score 358; lod 38		DHS
rs12959858	73,034,322	1	1	G	0.03	32kb 3' of TSHZ1	intergenic					FibroP,PanIslets			CACD,EBF,Irf	5			
rs35181847	73,037,586	1	1	C	0.03	36kb 3' of TSHZ1	intergenic								7 altered motifs				
rs12963413	73,039,236	1	1	C	0.03	37kb 3' of TSHZ1	intergenic								7 altered motifs	6			
rs4453592	73,040,883	0.85	1	T	0.02	39kb 3' of TSHZ1	intergenic								Rad21,Znf143,p300				
rs34468146	73,047,740	0.95	1	T	0.03	46kb 3' of TSHZ1	intergenic					Osteobl			Gm397,Mtf1	5			DHS, TFP(SRF)
rs71359085	73,048,182	0.95	1	G	0.03	46kb 3' of TSHZ1	intergenic					Medullo			5 altered motifs	5			DHS
rs34240979	73,052,310	0.95	1	C	0.03	50kb 3' of TSHZ1	intergenic								12 altered motifs	5			
rs12953745	73,053,195	0.66	1	C	0.02	51kb 3' of TSHZ1	intergenic					Th1,Medullo			Irf,SIX5	5			DHS
rs71359086	73,053,690	0.95	1	C	0.03	52kb 3' of TSHZ1	intergenic								5 altered motifs	5			
rs12956810	73,058,905	0.95	1	C	0.03	57kb 3' of TSHZ1	intergenic					H1-hESC			RXRA	5			

Annotation as in Supp. Table 2.

Supplementary Table 5. Tests for excess sharing of potential predisposition SNPs by CRC and EC cases at various statistical thresholds

		Endometrial Cancer					
		**$P<1x10^{-3}$**	**$P\geq1x10^{-3}$**	**$P<1x10^{-2}$**	**$P\geq1x10^{-2}$**	**P<0.05**	**P≥0.05**
**Colorectal Cancer**	**$P<1x10^{-3}$**	1	435	8	428	38	398
	**$P\geq1x10^{-3}$**	420	246040	3361	243099	14957	231503
		P=0.766		P=0.397		P=0.0208	
	**$P<1x10^{-2}$**	7	3346	50	3319	224	3129
	**$P\geq1x10^{-2}$**	414	243129	3303	240224	14771	228772
		P=0.589		P=0.524		P=0.138	
	**P<0.05**	24	14448	193	14279	913	13559
	**P≥0.05**	397	232027	3176	229248	14082	218342
		P=0.888		P=0.524		P=0.222	

*χ2 tests in genome-wide enrichment using 246,896 SNP with r2<0.1

No SNPs had p-values of less than 10^{-4} for both CRC and EC

Supplementary Figure 1. Association plots of *TSHZ1* region by CRC, EC and combined

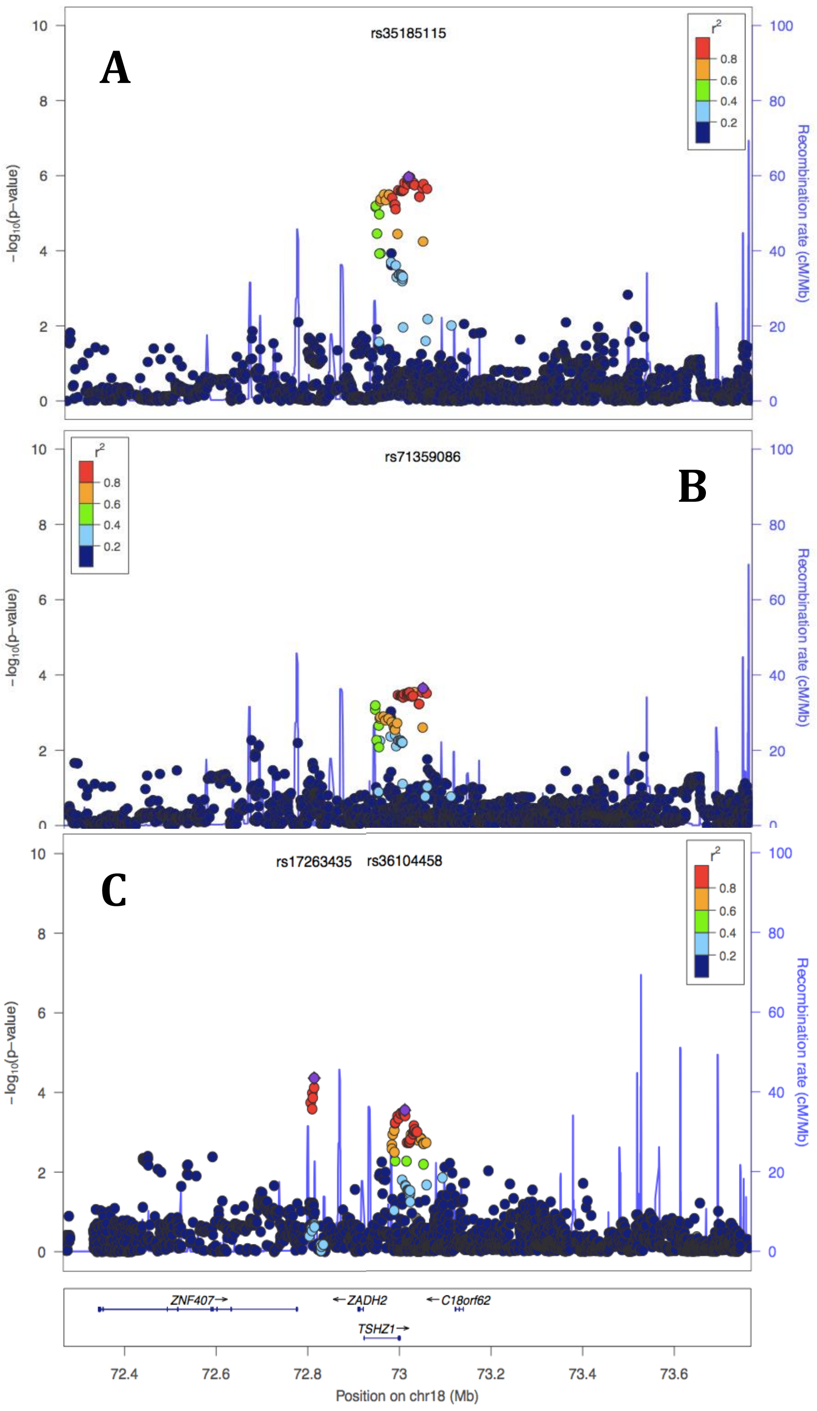

Regional association plots of TSHZ1 region using **A** CRC and EC GWAS **B** CRC GWAS **C** EC GWAS. rs12970291 ($P_{CRC/ECmeta}$=4.82×10^{-8}, top SNP typed in iCOGS for CRC and ERC analysis), rs35185115 ($P_{CRC/ECmeta}$=1.08×10^{-6}, top SNP typed or well-imputed in EC and CRC GWAS), rs71359086 (P_{CRC}=2.24×10^{-4}, top SNP in CRC GWAS), rs36104458 (P_{EC}=4.35×10^{-5}, top SNP in EC GWAS) all lie between 10.5 - 51.8kb downstream of TSHZ1, and are in perfect LD (r^2=1). The similarity of these three plots suggests that there may be a single causal variant downstream of TSHZ1 present in EC and CRC acting in opposite directions. An additional peak, rs17263435 (P=4.35×10^{-5}) is observed in the EC GWAS, but is not significant in CRC (P=0.10) . rs17263435 is not typed on iCOGS and has a CRC and EC meta-analysis p-value of 2.21×10^{-4}.

Supplementary Figure 2. LD and haplotype structure at TERT-CLPTM1L locus

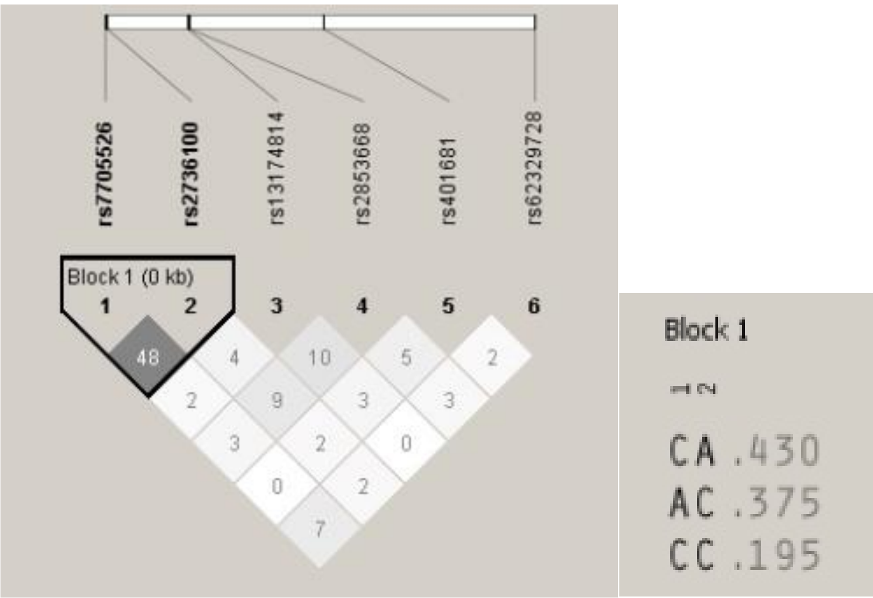

Plots were produced using HaploView for NSECG samples, showing pairwise LD (r^2) with standard colouring, left, and predicted haplotypes, right. SNPs were selected on the basis of reported associations from the literature with EC and/or CRC risk, albeit not a genome-wide levels of significance. rs2736100 was associated with CRC (Kinnersley, 2012). rs401681(Rafnar, 2009) and rs2853668 (Peters, 2009) have also been shown to be associated with CRC in previous studies of smaller scale. rs7705526, rs13174814 and rs62329728 have been recently associated with EC (Carvajal-Carmona 2014).
